# The Nottinghamshire Neurodiversity Network: building a multidisciplinary team of experts to improve pathways to care

**DOI:** 10.3389/frhs.2026.1792930

**Published:** 2026-03-18

**Authors:** Blandine French, Madeleine Groom

**Affiliations:** Institute of Mental Health, School of Medicine, University of Nottingham, Nottingham, United Kingdom

**Keywords:** care access, care pathway, healtcare provision, network, neurodevelopmental conditions

## Abstract

**Background:**

Neurodevelopmental conditions (NDCs), including attention-deficit/hyperactivity disorder (ADHD), autism spectrum disorder (ASD), and tic disorders, are highly prevalent across the lifespan and are associated with substantial unmet need within publicly funded health systems. In the UK, rapidly rising referral rates, combined with longstanding workforce and training gaps, have placed neurodevelopmental care pathways under constant pressure. Fragmentation across health, education, and social care has further limited services’ ability to respond coherently, resulting in prolonged waiting times, inequitable access, and avoidable adverse outcomes for individuals and families.

**Innovation:**

In response to these challenges, the Nottinghamshire Neurodiversity Network (NNN) was established in 2023 as a locally embedded, multidisciplinary stakeholder network. Its purpose was to translate research evidence and lived experience into implementable improvements across neurodevelopmental care pathways through sustained collaboration, co-production, and knowledge exchange.

**Approach:**

This community case study describes the rationale, context, design, and implementation of the NNN. It documents how the network was created, how stakeholders were engaged and organised, and how activities were structured to support pathway development, service evaluation, training, and policy engagement. Reflections on early impacts, lessons learned, and constraints are provided.

**Conclusions:**

The NNN shows how a locally embedded network can bring sectors together, clarify shared pathway problems, and support practical improvements in care. While context-specific, the principles underpinning the network's design and operation are transferable and may inform similar initiatives seeking to improve neurodevelopmental care through collaborative, system approaches.

## 1 Introduction: description of the nature of the problem and rationale for the proposed innovation

Neurodevelopmental conditions (NDCs), such as ADHD, autism spectrum disorder (ASD), and chronic tic disorders, affect approximately 8%–10% of the population (1–3). and frequently persist into adulthood (4). When timely diagnosis and appropriate support are unavailable, individuals with NDCs experience increased risks of poor mental and physical health outcomes, reduced educational and occupational attainment, and diminished quality of life (5). These impacts extend beyond individuals, affecting families, schools, workplaces, and publicly funded services (5, 6).

Over the past two decades, the UK has seen a marked and significant increase in referrals for neurodevelopmental assessment, particularly among adolescents and adults (7). This growth reflects increased awareness, changing diagnostic practices, and greater recognition of unmet need, especially among groups historically under-identified (8). However, service capacity has not expanded at a comparable rate. Publicly funded services face significant workforce shortages, limited training opportunities, and variable commissioning arrangements, resulting in long waiting lists, inconsistent eligibility thresholds, and fragmented pathways across the lifespan (9, 10).

A further challenge lies in the persistent gap between research evidence, policies, and everyday service delivery. Although there is a substantial evidence base demonstrating the benefits of early identification, coordinated care, and ongoing support for people with NDCs (11, 12), local systems often lack the infrastructure and cross-sector relationships needed to translate this knowledge into practice (13, 14). Responsibility for care sits across NHS providers, primary care, education, local authorities, and the third sector, each operating within different governance and funding arrangements.

In response to these urgent and unmet clinical needs, the Nottinghamshire Neurodiversity Network (NNN) was conceived as a locally responsive innovation. Rather than constituting a single intervention or service redesign, the network aimed to create a long-standing collaborative infrastructure through which stakeholders could jointly identify priorities, share knowledge, co-produce solutions, and support implementation (15, 16). Improving neurodevelopmental pathways requires more than technical solutions; it depends on sustained relationships and shared understanding across services.

## 2 Context: setting and population in which the innovation occurs

The NNN operates in Nottingham and Nottinghamshire, a region in the East Midlands of England that encompasses a mix of urban, suburban, and semi-rural communities with diverse socioeconomic characteristics. Neurodevelopmental services in this area are delivered across multiple NHS provider trusts and community settings, alongside education services, local authority provision, and a vibrant third sector. As in many regions, pathways for children, young people, and adults have developed separately over time, contributing to discontinuities, particularly at transition points.

It is also important to recognise the growing emphasis on needs-led support that is not contingent upon access to a diagnostic assessment, particularly given the strain on healthcare services. For example, in school settings, there may be several children with overlapping but distinct neurodevelopmental profiles, making environmental and structural adaptations—such as inclusive classroom practices an important part of the overall provision. These approaches may improve outcomes for individuals. They could also be essential in the current context where demands on services are escalating, reducing competition for scarce provision rather than causing further pressures on the system. They do, however, also rely on breaking down structural barriers and siloed ways of operating, which is a clear and important focus of the NNN.

Nationally, while extremely valuable (15, 16), stakeholder engagement in neurodevelopmental care is largely episodic, geographically focused, and issue-specific. While pockets of good practice exist (17), there are limited opportunities for system-wide dialogue about shared challenges, duplication, gaps in provision, or alignment between commissioning priorities and lived experience. Locally in Nottinghamshire, families and service users often reported uncertainty about roles and responsibilities across services, particularly when awaiting assessment or during transitions between child and adult services (18, 19).

Prior to the establishment of the network, we did not carry out a system-wide evaluation, but key stakeholders in the network shared findings from service data, commissioning discussions, and parent feedback which all highlighted concerns about fragmentation, waiting times, and poor communication, which inspired the development of NNN. The network targets the care pathway for children, young people, and adults with neurodevelopmental conditions, as well as their families and carers. Its stakeholder base intentionally spans healthcare professionals across disciplines, commissioners, service managers, researchers, educators, voluntary sector organisations, digital health innovators, and individuals with lived experience. This breadth reflects the network's explicit commitment to whole pathway thinking and to addressing neurodevelopmental care as a shared system responsibility rather than the remit of any single service.

## 3 Detail to understand Key programmatic elements

### 3.1 Establishment and governance of the network

The NNN was established in 2023 by academic clinical researchers based at the University of Nottingham, initially supported by institutional funding. From the outset, the network was positioned as an ongoing stakeholder collaboration rather than a time-limited research project. The network was positioned as a forum for joint problem-solving rather than consultation tied to a single project.

Initial stakeholder recruitment drew on existing professional and community networks across healthcare, education, commissioning, research, and the voluntary sector, supplemented by snowball sampling. An inaugural in-person conference and workshop in May 2023 served as both a launch event and an opportunity to establish shared priorities, clarify expectations, and agree on preferred modes of engagement. From the outset, neurodivergent individuals, family representatives, community groups, and charities were invited to and included in the steering group, contributing to priority setting, workshop discussions, and early decisions about the network's focus and format.

Power dynamics were recognised but not addressed through a formal framework. Trust developed gradually through repeated engagement and consistent framing of the network as a neutral, improvement-oriented space rather than a forum for critique or performance management. Clear notes and actions arising from each meeting and workshop were shared with all attendees, supporting this process. As trust-building was relational and emergent rather than a discrete intervention.

To support coordination while maintaining broad participation, a governance structure was developed that balanced strategic oversight with inclusive engagement. A small core leadership group coordinates overall direction and external liaison. The core group was established by the founding academic and clinical leads, with additional members invited based on expertise and leadership in service delivery, commissioning, research, and lived experience. The group includes both neurodivergent and neurotypical members and was formed to reflect multidisciplinary perspectives rather than diagnostic status alone. A larger multidisciplinary steering group meets regularly to identify priorities, guide activities, and facilitate translation into service contexts; this group includes clinicians, commissioners, researchers, and representatives with lived experience. Beyond this, a wider group of local members contributes through events, consultations, and working groups, while a broader professional network engages primarily through communications and larger knowledge-exchange activities.

This structure allowed people to engage at different levels while keeping decision-making clear. It also reflects the network's emphasis on relations and coordination across sectors, recognising that efficient pathway improvement requires both strategic alignment and distributed participation.

### 3.2 Engagement, communication, and knowledge exchange

A central aim of the NNN has been to create regular opportunities for cross-sector dialogue. Between May 2023 and December 2025, the network convened five in-person conferences, each combining research presentations, service updates, and facilitated workshops. These events were complemented by interim strategic workshops and online meetings, enabling momentum between larger gatherings.

**Figure 1:**
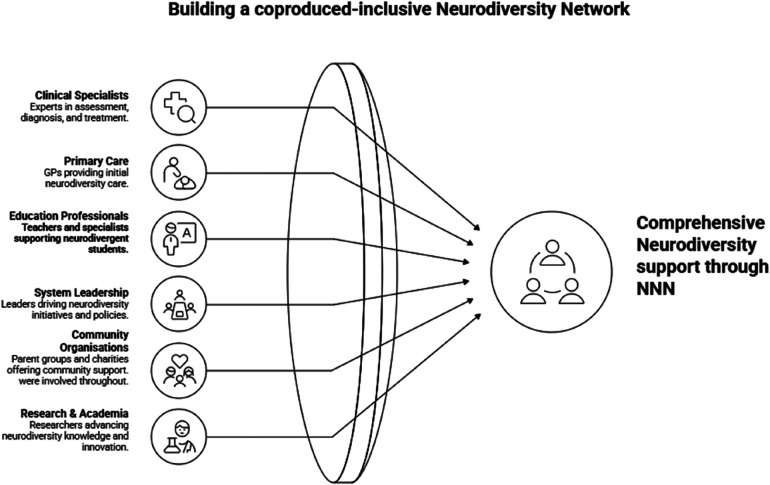
Description of the different roles in the Nottingham neurodiversity network steering group.

Workshops were designed to prioritise shared problem definition and practical outputs. Facilitated small-group activities enabled stakeholders to articulate barriers and facilitators to effective care, map existing pathways, and identify areas where change was both needed and feasible. These activities deliberately placed lived experience alongside professional expertise, reinforcing the principle that pathway design should be informed by those who use services as well as those who deliver and commission them (Figure 1).

To support ongoing communication, the network developed a dedicated website (20) providing a central hub for information, resources, and updates, alongside regular newsletters and targeted communications (3,951 visits since creation in 2024). This infrastructure helped to maintain engagement across a large and diverse membership. While a formal co-production framework was not systematically applied to all knowledge exchange activities, the design and delivery of meetings were informed by the network lead's lived experience and over a decade of facilitating parent and adult support groups, alongside ongoing feedback from neurodivergent individuals and families involved in NNN.

### 3.3 Co-production, service evaluation, and training

Co-production was embedded as a guiding principle of the network's activities. One example was a service evaluation exploring the experiences of parents whose children were on waiting lists for neurodevelopmental assessment. An evaluation of parents using children's services (*n* = 256), conducted in March 2024, examined how families could be better supported along the care pathway and identified consistent priorities. The most frequently reported concerns related to prolonged waiting times, uncertainty about referral status, and difficulty navigating fragmented services. When asked about improvements, parents most commonly prioritised regular updates on referral progress at one- to two-month intervals, preferably via email or letter, alongside clearer information about expected waiting times and what would happen at each stage of the assessment process. Families also highlighted the need for accessible local support relating to education, parenting, and neurodiversity while awaiting formal assessment. These findings directly informed the subsequent co-design workshop and changes to communication practices (full report (18).

Findings from the survey were taken forward into a facilitated co-design workshop with parents, supported by an external NHS co-design organisation, which generated practical, action-oriented recommendations including improvements to communication practices and the development of accessible pathway information. A key tangible outcome was the regional implementation of the Mind of All Kinds website (21), designed to provide structured, accessible information and interim support for families while awaiting assessment. The survey had identified uncertainty about referral status, lack of clarity regarding pathway stages, and limited access to local support as major concerns; the website was developed directly in response to these priorities and now serves as a regional resource offering guidance on assessment processes, signposting to local services, and support during waiting periods. Although formal impact evaluation has not yet been undertaken, the integration and continued use of the website within local NHS practice represent a concrete service-level output arising from stakeholder feedback and illustrate how structured engagement translated into implementable pathway improvements within existing commissioning arrangements.

In parallel, the network supported the co-development and dissemination of targeted training initiatives informed by both stakeholder priorities and research evidence. These included education-focused sessions addressing classroom-based neurodiversity support, training for general practitioners to strengthen confidence in identification and referral processes, and workshops for families navigating assessment pathways. In addition, collaboration with Health Innovation East Midlands supported the development of an evidence-informed Network Maturity Matrix and a structured business case for sustainability. This work is informing decisions about regional collaboration across Integrated Care Boards and helping to articulate the network's value proposition within existing commissioning frameworks, strengthening the potential for longer-term integration into NHS planning structures.

## 4 Discussion: practical implications and lessons learned for future applications

This community case study offers reflections for other regions and other healthcare services seeking to improve care pathways through collaborative approaches. Over two years, regular and structured engagement created opportunities for trust and shared understanding across sectors that are rarely achieved through one-off interactions. Sustained engagement built and maintained relationships between stakeholders, which also fostered honest discussion about the challenges experienced by providers of services, their ambitions for changes, and helped identify aspects of service delivery where improvement was feasible.

The network's governance structure appeared to support progression from dialogue to action. In particular, the steering group functioned as an interface between lived experience, frontline service delivery, and commissioning perspectives, helping to ensure that discussions were grounded in operational realities and that stakeholder input informed tangible adjustments, for example, improving communication with families on the waiting list for ND assessment.

Local data, combined with wider research evidence, provided a common reference point for discussion to ensure decisions were made with reference to a larger evidence base. Stakeholders reported finding this valuable, while acknowledging the constraints of existing commissioning and workforce structures.

Attention to stages of the pathway that are often overlooked—particularly waiting periods, communication, and transitions—highlighted opportunities for improvement that did not require full service redesign. While modest in scope, these adjustments were perceived as meaningful by families and service providers alike, reinforcing the importance of balancing feasibility with ambition.

It is important to note, as outlined in Section 6, this paper does not present formal effectiveness data or causal evaluation. Observations are derived from documented activities and stakeholder feedback rather than controlled outcome measurement. Even without formal evaluation, this case shows that dedicated coordination time and sustained cross-sector contact can support incremental change. Any application of these principles elsewhere would need to be adapted to local governance arrangements, resource constraints, and commissioning structures.

Formal outcome evaluation has not yet been undertaken; however, activities to date—including translating parent survey findings into service-level outputs, developing regional support resources, and establishing sustained cross-sector forums—demonstrate that the network has generated observable practice developments, which will require future structured evaluation to assess their effectiveness within existing system constraints.

Looking ahead, the network's priorities include consolidation, scalability, and sustainability. Current work involves mapping its development using a Network Maturity Matrix to assess progress, clarify areas for strengthening, and guide strategic planning. This process is informing decisions about regional collaboration, alignment across Integrated Care Boards, and approaches to securing sustainable funding to ensure the network can continue without relying on piecemeal funding and goodwill.

## 5 Acknowledgement of conceptual and methodological constraints

As a community case study, this paper does not present formal effectiveness data or causal evaluations. The NNN shows how a locally embedded network can strengthen cross-sector relationships and clarify shared challenges along the pathway, supporting practical improvements in care. However, these activities were developmental rather than investigator-driven research; while early impacts are encouraging, they cannot be attributed solely to the network or assumed to be generalisable without adaptation.

The NNN benefited from academic leadership, initial institutional funding, and pre-existing regional relationships. Network-based approaches are also resource-intensive, relying on ongoing coordination, leadership time, and stakeholder goodwill. Without continued support, there is a risk of burnout or uneven participation, potentially privileging stakeholders with greater capacity to engage.

Ethical considerations include managing expectations about the pace and scope of change, ensuring transparency about decision-making, and maintaining meaningful involvement of people with lived experience as the network grows.

## 6 Conclusion

The Nottinghamshire Neurodiversity Network demonstrates how a locally embedded, multidisciplinary network can support collaboration, learning, and improvement across complex neurodevelopmental care pathways. While not a substitute for structural investment in services, such networks can play a complementary role in bridging evidence, policy, and practice. This case study highlights transferable principles for other regions while underscoring the importance of context, sustainability, and equity in network-led innovation.

## Data Availability

The original contributions presented in the study are included in the article/Supplementary Material, further inquiries can be directed to the corresponding author.
